# Innate immune memory: The evolving role of macrophages in therapy

**DOI:** 10.7554/eLife.108276

**Published:** 2025-11-14

**Authors:** Payal Damani-Yokota, Kamal Mohan Khanna

**Affiliations:** 1 Department of Microbiology, New York University Grossman School of Medicine New York United States; 2 Perlmutter Cancer Center, New York University Langone Health New York United States; Queen's University Canada; National Institute of Immunology India

**Keywords:** macrophage, monocyte, trained immunity, innate immune memory, innate immunity, lung, bone marrow, infection

## Abstract

Trained immunity is reshaping our understanding of host defense by demonstrating that innate immune cells once thought to lack memory can be reprogrammed to mount heightened responses to subsequent challenges. Unlike tolerance, differentiation, or priming, trained immunity relies on epigenetic and metabolic rewiring of resident myeloid cells, particularly in mucosal barriers such as the skin, gut, and lungs, where these cells provide continuous protection against toxins and pathogens. Here, we review recent advances showing how an initial stimulus endows monocytes and macrophages with long-lasting functional changes that can be either protective or maladaptive upon re-exposure. We highlight therapeutic opportunities that harness trained immunity to boost vaccine efficacy and discuss strategies to modulate this program in cancer and hyper-inflammatory disorders. Finally, we propose new directions for enhancing or dampening trained immunity to promote human health.

## Historical perspective

The concept of immunological memory is well established, describing the phenomenon by which exposure to a pathogen enables the host to develop protective responses against subsequent encounters with the same pathogen —a foundational principle underlying vaccination (Ahmed and Gray, 1996; Plotkin, 2005). Macrophages play a pivotal role in innate immunity, offering the first line of defense against pathogens through rapid, non-specific responses. In 2007, Medzhitov and colleagues demonstrated that toll-like receptor (TLR) genes in murine macrophages bifurcated into two distinct functional categories following in vitro stimulation with lipopolysaccharide (Bukhbinder et al., 2022). These categories, marked by chromatin modifications, either silenced pro-inflammatory mediators or activated antimicrobial effectors, thereby showing adaptive-like properties in macrophages to regulate inflammation (Foster et al., 2007). Shortly after, Netea and colleagues introduced the concept of ‘trained immunity,’ defining it as the memory of the innate immune system, mediated by epigenetic and metabolic reprogramming, leading to enhanced responses upon secondary infections or stimuli (Netea et al., 2016). Since then, trained immunity has been observed in various innate immune cells, including macrophages, monocytes, natural killer (NK), neutrophils, and even hematopoietic stem cells (HSCs) in mouse and human studies (Netea et al., 2011; Saeed et al., 2014). Neutrophils, once thought too short-lived to ‘remember,’ can be centrally trained via reprogrammed granulopoiesis and emerge with enhanced antimicrobial and tissue-protective programs. β-glucan exposure, for example, generates trained neutrophils that promote disease tolerance to influenza and limit immunopathology, highlighting their relevance to barrier-tissue defense and vaccination strategies (Kalafati et al., 2023; Khan et al., 2025). Trained macrophages exhibit heightened functional capacities, including improved pathogen recognition, increased phagocytosis, and more robust cytokine production upon secondary exposure. Notably, ‘phagocytosis’ in the context of trained immunity is cargo-specific: recent work indicates that trained programs can differentially tune macrophage handling of distinct targets, with no change in antibody-dependent cellular phagocytosis (ADCP) but decreased efferocytosis of apoptotic neutrophils—an effect with direct implications for tumor immunity and resolution biology (Chatzis et al., 2025).

Since the formal designation of trained immunity, a series of studies have demonstrated that a primary infection with fungi such as *Candida albicans (C.albicans*) or an inoculation with a purified compound derived from fungal cell walls called β-glucan could induce long-lasting protection against subsequent heterologous challenges. Although not permanent, this protection resulted from epigenetic modification in monocytes following initial exposure, mediated by intrinsic activation of macrophage and monocyte PI3K/Akt/mTOR pathway (Quintin et al., 2012, Cheng et al., 2014). Findings from β-glucan studies challenged the conventional belief that immunological memory was exclusive to adaptive immunity offering new insights into human immune response to infection, environmental factors and vaccinations. Here, we provide a detailed account of recent advances in trained immunity in monocytes and macrophages, emphasizing developments particularly relevant to the lung.

Therapeutically, trained immunity offers significant potential in enhancing vaccine efficacy and modulating immune responses in disease settings. By harnessing the reprogramming of macrophages, we can improve host defense in the context of infections, as well as explore new avenues in cancer immunotherapy and autoimmune disease management. Central and peripheral training of macrophages, mediated by hematopoietic stem cells (HSCs) or tissue-resident populations, could provide innovative strategies to prevent or treat inflammatory and infectious diseases. This review explores the evolving role of macrophages in innate immune memory and their therapeutic implications in clinical settings.

## Central and peripheral training

**Figure 1.**
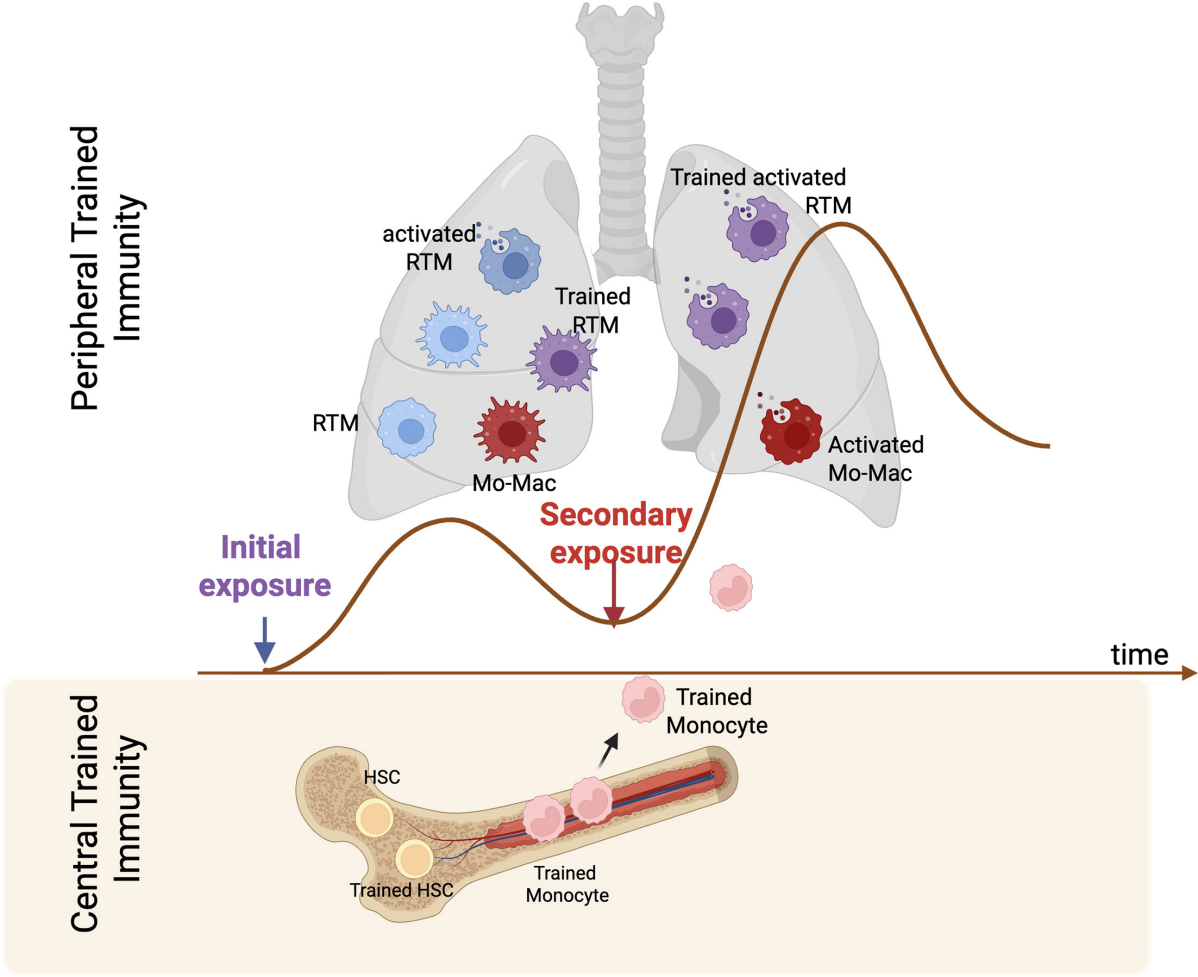
Central and peripheral trained immunity. A primary stimulus can initiate two complementary training programs. *Local (peripheral) training* occurs when resident tissue macrophages (resident macrophages RTMs, blue → purple with receptor activation) sense the insult, secrete cytokines, and acquire a trained phenotype within the lung. *Central (bone-marrow) training* arises when the same stimulus reprograms hematopoietic stem and progenitor cells (HSPCs) in the bone marrow, producing primed monocytes that enter the circulation, migrate to the lung, and differentiate into trained monocyte-derived macrophages (Mo-Macs, red). For clarity, we define central trained innate memory (CTM) as training strictly within HSPCs/myeloid progenitors. Peripheral trained innate memory (PTM) encompasses local training of mature macrophages, including those residing in bone marrow niches. These pathways may act independently or together, depending on the nature, timing, and intensity of the initial insult. Upon re-exposure, whichever trained population is present mounts an enhanced, coordinated response, illustrating that peripheral and central pathways are bona fide and often cooperative forms of innate immune memory. Image generated using BioRender.com.

Trained immunity manifests both in the bone marrow (Sohrabi et al., 2020), where it involves hematopoietic stem and progenitor cells (HSPCs), known as ‘central trained innate memory (CTM),’ and in fully differentiated, mature innate immune cells or tissue-resident cells within peripheral tissues, referred to as ‘peripheral trained innate memory (PTM).’ In this review, we define CTM strictly as training that occurs at the level of HSPCs in the bone marrow, leading to durable imprinting of their myeloid progeny. By contrast, when we discuss macrophages resident within the bone marrow niche (non-HSPC compartment), we consider these as peripheral/local training events occurring in a lymphoid organ or mucosal tissues. A substantial body of evidence supports the concept of CTM, particularly from studies demonstrating trained immunity in HSCs, myeloid progenitors, and BM–derived monocytes following infections or immunization (e.g. with BCG) (Cirovic et al., 2020; Kaufmann et al., 2018; Mitroulis et al., 2018). In contrast, peripheral or local trained immunity, where tissue-resident macrophages undergo long-term functional reprogramming at the site of infection or inflammation remains less well understood (Netea et al., 2020). Therefore, for a comprehensive understanding of trained immunity, parallel investigation is necessary to understand the impact of primary stimuli in the BM as well as peripheral tissues that may collectively contribute to host immune consequences, as shown in an example in Figure 1, where trained immunity is orchestrated in the lungs locally as well as via BM-derived precursors.

To date, there is limited evidence describing the mechanisms by which tissue-resident macrophages mediate PTM, which is defined as their capacity to confer protection against secondary challenges independently of BM-derived cells. One major barrier to advancing this field is the lack of selective experimental tools and models that can deplete or manipulate specific tissue-resident macrophage populations without affecting other myeloid cells. Traditional approaches, such as clodronate liposome administration to deplete macrophages have many nonspecific effects on other myeloid cells, such as neutrophils (Culemann et al., 2023; Mass, 2023). More recently, transgenic mouse models in which human diphtheria toxin receptor (DTR) is selectively expressed on immune cells (e.g. CD11b-DTR, CD169-DTR, or NAM-DTR) (CD169-cre mice crossed to CX3CR1-flox-DTR) mice have been used to deplete lung interstitial macrophages and broader tissue-resident macrophage populations (Ural et al., 2020; Hua et al., 2018; Yu et al., 2023; Gaertner et al., 2024; Borthwick et al., 2016; Duffield et al., 2005; Miyake et al., 2007). However, even these tools lack the resolution to distinguish among distinct macrophage subsets within tissues, such as in the lung. Nevertheless, recently, several new more sophisticated transgenic mice have been developed that have allowed investigators to probe functions and ontogeny of specific subsets of macrophages. These include CX3CR1-Cre/Ert2-Cre (allows for lineage tracing and targeting of tissue resident macrophages) (Yona et al., 2013), Ms4a3-Cre (allows lineage tracing of bone marrow-derived macrophages in tissues) (Liu et al., 2019) and the split-Cre (*Lyve1^ncre^:Cx3cr1^ccre^;* binary transgenic Cre approach to dissect functions of specific subsets of macrophages) (Boura-Halfon et al., 2024). Thus, more precise transcriptomic profiling is needed to identify unique surface markers and regulatory pathways that govern the maintenance and function of resident macrophage subsets (Hua et al., 2018).

To understand the broader implications of trained immunity, it is essential to compare the mechanisms involved in central versus peripheral training, particularly within macrophages across various tissues. While CTM involves systemic changes in HSC-like progenitors leading to enhanced immune responses in peripheral tissues, PTM directly involves epigenetic modifications within tissue-resident macrophages exposed to local pathogens. This distinction is particularly important when analyzing trained immunity across lymphoid and mucosal tissues, as the nature of training in these distinct environments may vary based on local immune challenges and the specific tissue microenvironments involved. An important unresolved question is how turnover dynamics in mucosal macrophage pools influence the relative weight of central vs peripheral training. In tissues like the lung and gut, where resident populations are rapidly replaced by BM-derived precursors, sustained trained immunity likely relies more on central training. For example, the alveolar macrophages (AMs) are known to rapidly undergo cell death following infections and are replaced by bone marrow-derived monocytic precursors (Li et al., 2022b; Aegerter et al., 2020; Hartung and Esser-von Bieren, 2022). However, other lung resident macrophages, such as a subset of interstitial macrophages (IMs) known as the nerve and airway-associated interstitial macrophages (or NAMs) that proliferate after infections may exhibit local training (Ural et al., 2020). Similarly, in organs such as the brain or liver, long-lived resident macrophages may maintain peripheral training for extended periods (Waisman et al., 2015).

The interval between the primary stimulus and detection of trained phenotypes varies with the cellular locus of training and the stimulus used. CTM (HSPC-level) training is typically assessed weeks to months after exposure and can persist long-term because HSPCs continuously generate primed myeloid progeny (e.g. Bacillus Calmette–Guérin (BCG) and β-glucan remodel HSPCs and myeloid progenitors). Peripheral training of mature macrophages is often assayed days to weeks post-exposure and tends to wane with local turnover, especially in mucosal sites. In the lung, influenza and helminth models reveal training detectable weeks later, with durability depending on whether resident pools are replaced by BM-derived cells. Notably, human and animal studies suggest innate memory can persist up to ~1 year in some contexts, consistent with an HSPC reservoir of epigenetically imprinted progenitors (Netea and Joosten, 2024a; Mhlanga et al., 2025).

## Macrophage training across different organs and tissues

**Figure 2.**
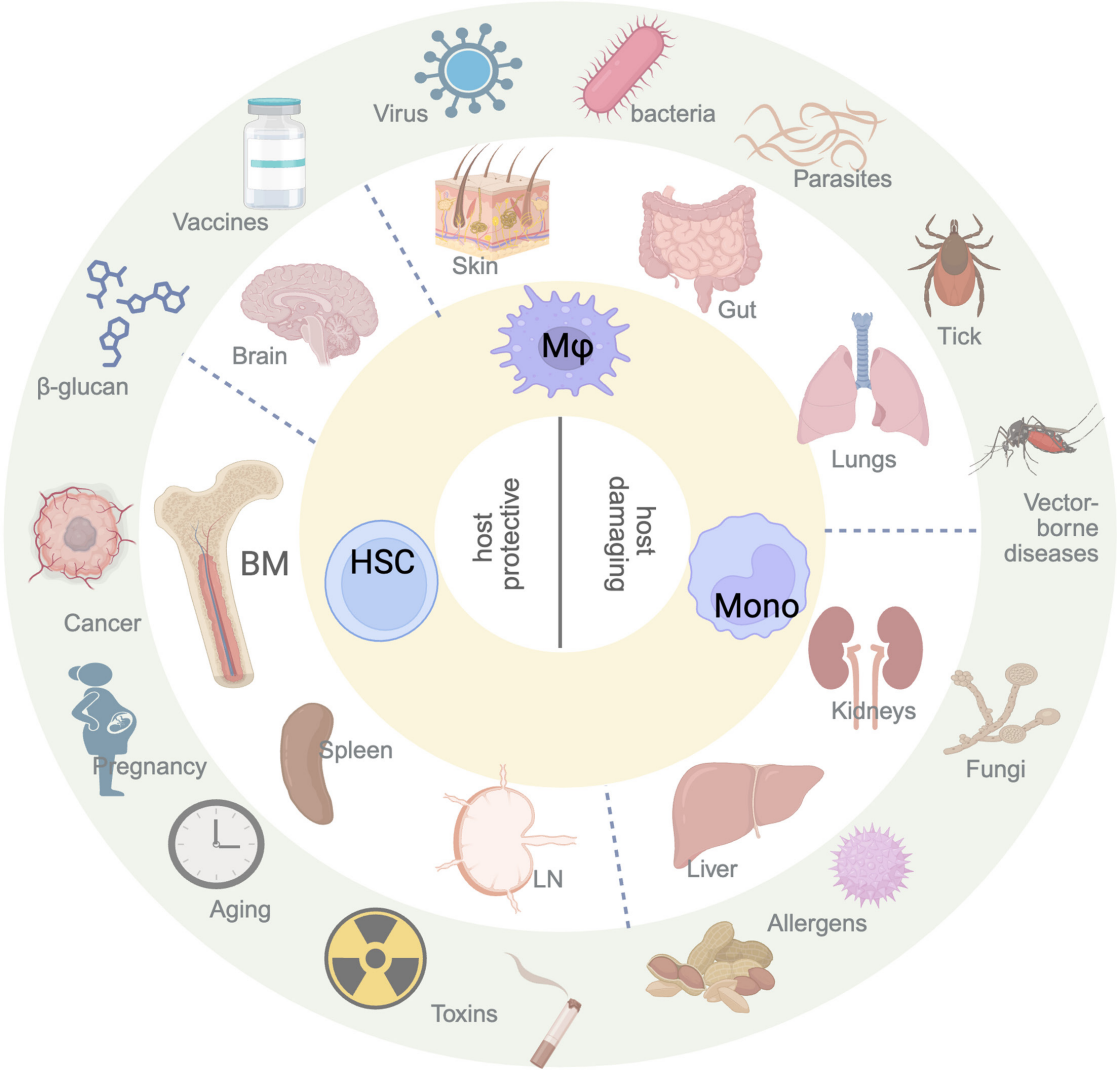
Triggers of trained immunity across all tissues. Peripheral and central trained immunity can be initiated by diverse stimuli, including pathogens (viruses, bacteria, fungi, vector-borne agents, and enteric or blood parasites), allergens (food- or environmental-derived), purified microbial or synthetic compounds, vaccines, toxins (radiation, air pollution, cigarette smoke), physiological processes such as aging and pregnancy. Following the primary stimulus, macrophages in mucosal sites (skin, gut, lung), lymphoid organs (spleen, bone marrow, lymph nodes), lymphatic-associated tissues (liver, kidney), and the brain undergo profound epigenetic and metabolic reprogramming. These trained macrophages and the monocytes they give rise to subsequently mount an amplified immune response to secondary challenges, which can be host-protective or host-damaging depending on context. Image generated using BioRender.com.

Frequency of macrophages varies vastly in mucosal tissues such as the gut, skin, and lungs compared to lymphoid tissues, despite a presence of tissue-resident macrophages in all. Macrophages in the lymphoid tissues, such as the spleen and the BM exhibit a wide variety of phenotypes and functions such as removal of apoptotic cells to thereby prevent autoimmunity, as well as kill cancer cells and activating other adaptive as well as innate cells (den Haan and Martinez-Pomares, 2013). Whereas the primary purpose of the mucosal tissues is to serve as a barrier between the body and the external environment, these tissues have profound tissue-specific secondary functions (Dickson et al., 2025). Due to a constant exposure to pathogens, allergens, and environmental pollutants, macrophages in the lungs (AMs), skin (dermal macrophages) as well as the gut (intestinal macrophages) undergo trained immunity for a rapid, localized responses to secondary infections (Li et al., 2022b; Aegerter et al., 2020; Chen et al., 2023b; Feuerstein et al., 2020; Leopold Wager and Schlesinger, 2025; Zahalka et al., 2022; Chen et al., 2022a; Misharin et al., 2017; Wang et al., 2023). Additionally, the plasticity of mucosal macrophages allows them to enhance their immune functions, such as cytokine production and phagocytic activity, in response to repeated exposure to similar pathogens. Moreover, they also exhibit the ability to modulate tissue repair and homeostasis, particularly in the lungs, where macrophages play a crucial role in tissue regeneration after injury caused by pathogens or environmental toxins. In this section, we present a thorough comparative analysis of trained immunity of macrophages in lymphoid vs mucosal tissues. Figure 2 shows a comprehensive and diverse array of stimuli that trigger trained immunity in the myeloid progenitors (Sohrabi et al., 2020) and macrophage populations (BM and periphery) across all tissues that result in host beneficial or host damaging consequences.

## Lymphoid tissues

Trained immunity in lymphoid organs critically shapes host defense. When macrophages in the bone marrow, spleen, and lymph nodes acquire a trained phenotype, they respond more rapidly to early danger signals, speeding pathogen clearance and priming adaptive immunity. Yet this heightened reactivity can turn maladaptive, fueling excessive inflammation that underlies autoimmune and other chronic inflammatory conditions. The sections that follow offer an overview of how trained immunity reprograms macrophages in the bone marrow, spleen, and lymph nodes.

### Bone marrow

Here, we specifically discuss bone marrow–resident macrophages (not HSPCs), which can undergo local or peripheral training within the marrow niche. This distinction separates CTM at the progenitor level from training of mature macrophages that reside in BM. In bone marrow-derived macrophages (BMDMs), inflammatory cues, particularly IL-1β and microbial products trigger epigenetic remodeling, including histone acetylation and changes in methylation status. BM-derived macrophages mount vigorous responses to systemic infections and play a pivotal role in maintaining long-term immune memory after vaccination (Netea and Joosten, 2024b; Netea et al., 2016; Netea et al., 2020).

Exposure to β-glucan or BCG reprograms BM myeloid progenitors, imprinting trained immunity on their myeloid descendants. In mice, BCG administration remodels HSCs through IFN-γ–dependent myelopoiesis that confers broad protection against later infections (Kaufmann et al., 2018). β-Glucan expands CD41^+^ HSCs via IL-1β- and GM-CSF-dependent pathways, rewiring glycolysis and cholesterol biosynthesis. Such central training accelerates emergency myelopoiesis during infection but can also skew hematopoiesis in chronic inflammation (e.g. atherosclerosis, sepsis), where continuous production of primed myeloid cells worsens pathology (Netea et al., 2016; Netea et al., 2020).

Experimental infection with *Candida albicans* or exposure to β-glucan primes monocytes to mount stronger responses against secondary challenges via production of proinflammatory cytokines (Quintin et al., 2012; Marakalala et al., 2013; Brown and Gordon, 2003; Bashir and Choi, 2017). β-Glucan-driven training hinges on apolipoprotein E (ApoE) modulation of Dectin-1 signaling (Theobald et al., 2024), which, via IL-1β, stimulates proliferation of CD34^+^ hematopoietic stem cells and epigenetically reprograms their myeloid progeny (Mitroulis et al., 2018; Moorlag et al., 2020; Braian et al., 2023; Brueggeman et al., 2022; Camilli et al., 2020; Tannahill et al., 2013; Zhong et al., 2020). These modifications leave macrophages and monocytes poised for rapid, protective responses to heterologous pathogens.

BCG vaccination is another well-established inducer of trained immunity in both mice and humans. Beyond its efficacy against *Mycobacterium tuberculosis*, BCG lowers all-cause infectious mortality in infants (Chen et al., 2022a) and confers broad protection against pathogens ranging from *Bordetella pertussis* and *Schistosoma mansoni* to influenza virus and SARS-CoV-2 in animal models (Kaufmann et al., 2018; O’Neill and Netea, 2020; Spencer et al., 1977; Covián et al., 2019; Nascimento et al., 2008; Pittet et al., 2023; Gillard et al., 2022; Tribouley et al., 1978; van ’t Wout et al., 1992). Similar nonspecific benefits have been documented in humans (Biering-Sørensen et al., 2017; Rieckmann et al., 2017; Jensen et al., 2006; Lund et al., 2015; Benn et al., 2013; Kleinnijenhuis et al., 2014; Cime-Castillo et al., 2018; Walk et al., 2019; Stewart and Levine, 2011; Powles et al., 1977; Villumsen et al., 2009; Buffen et al., 2014). An early BCG vaccination can protect neonates from infant mortality by 43% for deaths related to infectious diseases. However, it does not significantly affect mortality from non-infectious causes. This suggests cross-protection through the priming of immune cells via trained immunity induced by BCG. For example, early BCG immunization reduces neonatal deaths from infectious diseases by 43%, and co-administration with smallpox vaccine correlates with a lower risk of HIV-1 acquisition, highlighting its potential to enhance future HIV vaccine strategies (Biering-Sørensen et al., 2017; Rieckmann et al., 2017; Jensen et al., 2006). Clinical trials also point to BCG-mediated protection in melanoma, leukemia, and lymphoma, although one study found that BCG worsened pathology following controlled malaria infection, underscoring the context-dependent nature of trained immunity (Stewart and Levine, 2011; Powles et al., 1977; Villumsen et al., 2009; Walk et al., 2019).

Other infections can similarly induce trained immunity: *Helicobacter pylori (H. pylori*) infection of primary monocytes enhance their response to subsequent *Escherichia coli (E. coli*) challenge as well as increased production of NF-kβ specific proteins such as p65/RelA leading to an enhanced response to an LPS challenge (Frauenlob et al., 2023); *S. aureus* primes macrophages for protection against *E coli* challenge (Carlile et al., 2024); and *Streptococcus pneumoniae* GM-CSF-dependent macrophage training for protection against diverse pathogens (Xu et al., 2024). Collectively, these findings highlight how trained immunity in myeloid cells shape subsequent infection outcomes via epigenetic and metabolic rewiring.

### Spleen

The spleen is the body’s largest secondary lymphoid organ that filters blood-borne pathogens and senescent cells while coordinating pathogen clearance and immune-cell activation and harbors many different subsets of macrophages in different compartments (Lewis et al., 2019, Perez et al., 2017, Mebius and Kraal, 2005). As in other tissues, red-pulp macrophages (RPMs) can likewise be trained: after BCG exposure, they protect mice against subsequent *Salmonella enterica* serovar Typhimurium infection (Solomon et al., 2025). Fate-mapping studies (MS4A3; MS4A3^Tdtm^; CX3CR1^GFP^) show that monocytes recruited to the RPM niche undergo IFN-γ/STAT1-dependent reprogramming; disruption of this pathway abolishes protection, underscoring the critical role of training within splenic myeloid compartments (Liu et al., 2019).

In β-glucan–primed mice, splenic macrophages and monocytes produced normal amounts of TNF-α and IL-6 after ex vivo TLR2 stimulation, yet splenectomy eliminated the neutrophil recruitment typically associated with trained immunity, highlighting the spleen as a critical, if under-appreciated, hub in this process (Ferreira et al., 2022). By contrast, splenic macrophages from BCG-vaccinated mice displayed hallmark features of training: permissive histone marks and up-regulation of CCR2, CXCR4, TLR2/4, Ly6C, CD40, CD43, CD68, CD80, CD206, MHC-II, and glycolytic activity (lactate production) after LPS restimulation (Jeljeli et al., 2019). Furthermore, BCG priming protects against secondary *Salmonella Typhimurium* challenge through STAT1-dependent reconstitution of both resident red-pulp macrophages and BM-derived monocytes, underscoring the coordinated roles of local and central trained-immunity pathways (Solomon et al., 2025).

### Lymph nodes

Lymph nodes are critical hubs of the lymphatic system, filtering lymph, presenting antigens, and coordinating innate and adaptive immunity. They develop during the first and second trimesters of pregnancy (Cupedo, 2011; Liao and von der Weid, 2015). Within the node, macrophages act as scavengers, sentinels, and trophic effectors, guiding distinct phases of the immune response (Bellomo et al., 2018). Although studies of trained immunity in lymph‐node macrophages remain limited, most focus on infection models that examine two key subsets. Subcapsular sinus macrophages (SSMs) line the entry sinuses, where they capture and archive lymph-borne pathogens and antigens. Medullary sinus macrophages (MSMs), situated deeper in the node, specialize in antigen processing and presentation (Gray and Cyster, 2012; Camara et al., 2022).

CD169^+^ macrophages require RANKL and lymphotoxin-β receptor signaling to establish their niche and differentiate (Camara et al., 2022; Cordeiro et al., 2016). Moreover, SSMs are highly efficient in exosome capture (Liu et al., 2021), and sequestering exosomes (Xu et al., 2024). During *Toxoplasma gondii* infection, NK cells accumulate in the subcapsular space, where NK cells form stable contacts with collagen fibers and CD169^+^ Ly6C^+^ SSMs suggesting that parasite-induced training of CD169^+^ SSMs helps activate NK cells and enhance host protection (Coombes et al., 2012).

Macrophages in skin-, lung-, and gut-draining LNs are crucial for clearing pathogens, yet their training potential is only beginning to be explored. In a murine house-dust-mite (HDM) induced asthma, prior infection with murid γ-herpesvirus 4 (MuHV-4) results in a reduced type-2 cytokine secretion via an increase in regulatory monocytes suggesting virus-trained mediastinal LN-derived macrophages can dampen asthma severity (Murphy et al., 2021). Additionally, a single influenza A virus infection causes lasting MedLN enlargement and better T-cell responses to unrelated subsequent infections, indicating durable regional reprogramming (Stolley et al., 2024).

Mesenteric lymph nodes (MLN) ferry bacteria to the gut and shape subsequent mucosal responses, triggering cytokine production characteristic of trained immunity (Cortes-Perez et al., 2021). Oral bacterial lysate likewise drives MLN expansion and broad activation of immune cells, again hinting at macrophage training (Navarro et al., 2011). Taken together, these studies show that LN macrophages, long viewed as gatekeepers of antigen capture and clearance, are now emerging as dynamic players in trained immunity, where BM-derived subsets can acquire epigenetic memory that reprograms a regional immune tone, influencing outcomes for asthma and cancer, offering new therapeutic opportunities.

## Mucosal tissues

**Figure 3.**
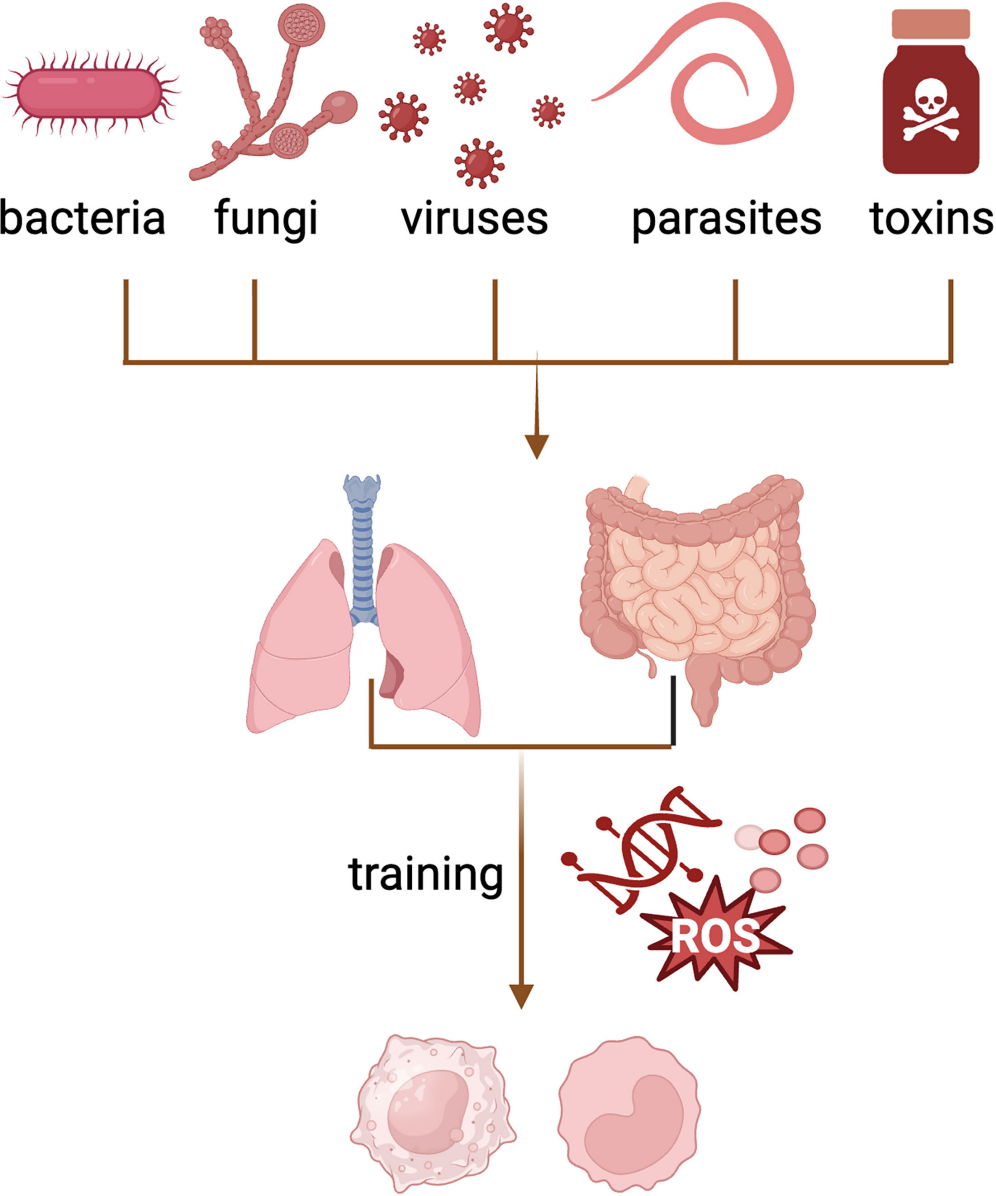
Trained immunity of macrophages and monocytes in mucosal tissues. Trained immunity in mucosal tissues is induced by a variety of stimuli causing local programming of tissue-resident macrophages via metabolic and epigenetic reprogramming resulting in long-term changes in macrophages and monocytes. Image generated using BioRender.com.

Mucosal barriers, including the skin, lungs, and gut are in constant contact with the external environment and thus serve as primary entry points for pathogens and toxins. In this section, we focus on triggers and consequences of trained immunity in mucosal tissues, such as the lungs and gut by highlighting both the resident macrophage and BM-derived precursors that repopulate mucosal tissues as shown in Figure 3.

### Lungs

Pathogen exposure is a key driver of trained immunity in lung macrophages enhancing functions of both tissue-resident macrophages (RTM) and BM-derived monocytes via long-term functional adaptations that can either enhance or impair the host’s ability to respond to subsequent challenges (Table 1). Mouse infection and vaccination models have been invaluable for determining whether this training enhances or, in some cases, impairs subsequent host defense.

**Table 1.** Summary of trained immunity in lung macrophages.

Stimulus / Exposure	Macrophage Subset(s)	Training Outcome	Duration / Turnover	Therapeutic / Pathological Implications	Relevant References
Influenza infection	AMs, Mo-AMs, IMs (incl. NAMs)	Enhanced IFN-γ/IL-1β signaling, metabolic & chromatin remodeling	Weeks; largely BM-derived AM replacement, NAMs expand locally	Protects against secondary bacterial pneumonia; context-dependent (resident vs BM-derived)	Ural et al., 2020; Li et al., 2022b; Aegerter et al., 2020; Banete et al., 2021; Vangeti et al., 2023; Hoeve et al., 2012; Iliakis et al., 2023
SARS-CoV-2 infection	AMs, Mo-AMs	Elevated CXCL10, TNF-α, IL-6 (trained immunity phenotype)	Persistent post-infection; risk of chronic inflammation	Contributes to antiviral defense but may drive post-acute COVID-19 syndromes	Yeung et al., 2025; Winkler et al., 2020; Cvetkovic et al., 2023; Netea et al., 2023; Netea and Joosten, 2023; Arunachalam et al., 2020; Machiels et al., 2018
Helminth infection (e.g., Nb, Sv, Hp)	AMs, Mo-AMs	Type 2 skewing (Arg1⁺, RELMα⁺), tissue repair phenotype	Lasting after infection; Mo-AMs>TR AMs in larvicidal activity	Enhances repair and tolerance; primes CD8⁺ T cells, cross-protection (e.g., SARS-CoV-2)	Aegerter et al., 2020; Chen et al., 2022a; Misharin et al., 2017; Bouchery et al., 2015; Sveiven et al., 2023; Weyand et al., 2017; Marsland et al., 2008; Silveira et al., 2002; Oliveira et al., 2019; Chen et al., 2012
Allergens (HDM) & viral co-infection (MuHV-4)	AMs, Mo-AMs, regulatory monocytes	Regulatory training reduces Th2 priming	Weeks–months	Protective against asthma exacerbations	Lechner et al., 2022
Environmental exposures (e.g., Borrelia, HDM)	Lung macrophages, BM-derived progenitors	Glycolysis-dependent memory-like states; TNF modulation	Variable	Protection vs parasites (Ascaris), but potential for maladaptive inflammation	Cheng et al., 2014
Vaccines (BCG, influenza, COVID-19)	AMs, BM-derived monocytes	Epigenetic & metabolic reprogramming	Months; reinforced by central training	Enhances heterologous protection; may synergize with cancer therapies	Cirovic et al., 2020; Mhlanga et al., 2025; Buffen et al., 2014; Netea et al., 2023; Bulut et al., 2025; Debisarun et al., 2021; Chakraborty et al., 2023; Gu et al., 2023; Principi and Esposito, 2024; Gurfinkel et al., 2004; Jurado et al., 2025

Throughout life, the lungs contend with a wide spectrum of infectious and non-infectious insults, gradually shaping immune memory within their myeloid compartment, especially among macrophages. Two principal tissue-resident populations occupy distinct niches: AMs in the airspaces and IMs in the lung parenchyma (Soos et al., 2006). Over the past decade, IMs have been subdivided into multiple subsets distinguished by surface markers, location, and function (Ural et al., 2020; Chakarov et al., 2019; Gibbings et al., 2017; Li et al., 2024). Using mouse models of respiratory viral infections with PR/8 (H1N1) influenza strain (Ural et al., 2020) and mouse-adapted SARS-CoV-2 (MA-10) infection (Yeung et al., 2025), we have shown that AMs are largely supplanted by incoming monocytes, while NAMs undergo a local expansion, continuing for several days to secrete regulatory cytokines and other factors that dampen inflammation and promote disease tolerance in the lung (Ural et al., 2020; Yeung et al., 2025).

Pathogen exposure imprints durable programs of trained immunity on lung macrophages. In mice, primary influenza infection activates IFN-γ and IL-1β pathways that drive long-lasting metabolic and chromatin remodeling in AMs, thereby strengthening resistance to secondary bacterial pneumonia (Aegerter et al., 2020; Banete et al., 2021). Human monocytes exposed to influenza antigens likewise produce more TNF-α and IL-6 upon restimulation, suggesting that this mechanism is conserved across species (Vangeti et al., 2023; Hoeve et al., 2012). Yet whether the trained AMs are bona fide tissue residents or BM-derived replacements remains debated: several murine studies, spanning influenza (Li et al., 2022b; Aegerter et al., 2020), *N*ippostrongylus brasiliensis (Nb) (Chen et al., 2022a) and cancer models (Wang et al., 2023) show that monocyte-derived AMs outperform resident AMs in heterologous defense, and a recent commentary by Iliakis et al. argues that post-infection AM pools are largely BM-derived in origin (Iliakis et al., 2023)*.* This controversy underscores the need for tools that can interrogate resident subsets, such as NAMs, which may expand locally rather than being largely replaced by BM- derived progenitors.

SARS-CoV-2 infection provides a second paradigm. In K18-hACE2 mice, infection with the WA01 strain induces trained immunity in lung macrophages through type I and II interferon signaling (Winkler et al., 2020). Similarly, human monocytes exposed to SARS-CoV-2 exhibit trained immunity characterized by elevated CXCL10 production upon restimulation (Cvetkovic et al., 2023), as well as increased TNF-α and IL-6 production. These responses contribute to protective immunity but may also heighten the risk of excessive inflammation observed in post-acute COVID-19 syndromes (Netea et al., 2023, Netea and Joosten, 2023). Single-cell and bulk transcriptomic analyses of severe-COVID cohorts reveal impaired type I IFN signatures and reduced HLA-DR on circulating myeloid cells (Arunachalam et al., 2020), highlighting pathways that could be targeted therapeutically. In a heterologous infection with primary infection of MuHV-4 replaces AMs with regulatory monocytes that blunt dendritic-cell priming of TH2 responses, thereby protecting mice from HDM-induced asthma (Machiels et al., 2018).

Helminths parasitic infections also reshape pulmonary innate memory. Lung-migrating parasites such as *Nb* and *Strongyloides venezuelensis* (*Sv*) trigger potent type 2 responses; group 2 innate lymphoid cells secrete IL-5, recruit eosinophils, and promote parasite killing and has an impact on clinical symptoms of chronic allergic diseases (McSorley et al., 2019; Bouchery et al., 2015; Yasuda et al., 2018; Gazzinelli-Guimaraes et al., 2019). These helminths damage lung tissue and skew macrophages toward an M2-like, Arginase-1– and RELMα-rich phenotype that accelerates tissue repair (Chen et al., 2022a; Bouchery et al., 2015; Sveiven et al., 2023; Weyand et al., 2017; Marsland et al., 2008; Silveira et al., 2002; Oliveira et al., 2019; Chen et al., 2012). Mo-AMs also demonstrate superior larvicidal activity compared to tissue-resident alveolar macrophages (TR-AMs), a capacity that persists after lung injury and infection (Aegerter et al., 2020; Misharin et al., 2017). Notably, prior infection with *Nb* enhances CD8 + T cell responses and protects against secondary SARS-CoV-2 infection in K18-hACE2 mice via macrophage priming (Hilligan et al., 2022). However, mechanisms by which parasitic infections induce trained immunity in macrophages or monocytes remain unknown.

In vitro, *Fasciola hepatica* extracts reprogram macrophages to secrete more IL-10 and IL-1Ra and less TNF-α and IL-12p40 following LPS or Pam3Cys restimulation (Quinn et al., 2019). Additionally, *Schistosoma mansoni* infection induces metabolic reprogramming of macrophages through mitochondrial biogenesis (Cortes-Selva and Fairfax, 2021; Cortes-Selva et al., 2021; Oviedo et al., 2025), while *Leishmania major* infection primes BM-derived macrophages via histone modifications and sustained glycolytic metabolism causing an increased TNF-α and IL-6 production upon heterologous bacterial challenge (Liu and Uzonna, 2012). Collectively, helminths induce trained immunity through diverse epigenetic and metabolic routes that enhance resistance to secondary type 1 infections.

Other environmental exposures, such as *Borrelia burgdorferi* infection produces a tissue-wide, glycolysis-dependent memory-like state in macrophages with reduced TNF-α (Barriales et al., 2021), whereas HDM exposure confers protection against *Ascaris* by reprogramming BM progenitors and increased CCL17 and cysteinyl-leukotriene via TNF-dependent priming of human monocyte-derived and murine progenitor cells (Lechner et al., 2022). Collectively, these findings highlight an essential role of type 2 immunity in mediating protection against secondary infections through trained immunity mechanisms involving macrophages and monocytes (Hartung and Esser-von Bieren, 2022; Cvetkovic et al., 2023; Bouchery et al., 2015; Yasuda et al., 2018; Mabbott, 2018; Silva Pereira et al., 2019; Ahrends et al., 2021).

### Gut

The gastrointestinal (GI) mucosa is a vast interface with the external environment, continually exposed to pathogens, dietary antigens, and toxins. Such encounters imprint long-lasting programs on gut macrophages, enhancing their capacity to clear microbes, regulate inflammation, and support tissue repair. Three broad factors drive this training: (1) chronic inflammatory disorders, including inflammatory bowel disease (IBD), Crohn’s disease (CD), and ulcerative colitis (UC); infections with enteric pathogens; and (3) immune dysregulation associated with altered microbiota composition and function disorders (Ordovas-Montanes et al., 2018; Velikova et al., 2024; Ramanan et al., 2016). Notably, excessive gut inflammation correlates with overproduction of IL-1β, TNF-α, IL-18, and IL-17 (de Mattos et al., 2015; Lu et al., 2022; Lee et al., 2018). Furthermore, experimental models of Crohn’s disease show that infection with enteric parasites confers protection in mice from intestinal abnormalities due to the lack of *Nod2* gene during infection with *bacterioides* species (Ramanan et al., 2016). These studies point to the critical importance of hygiene hypotheses and how trained immunity guides and reshapes immune landscapes for host beneficial consequences in the context of infection.

Oral administration of heat-killed *E. coli* modulates subsequent responses to *Salmonella* challenge, boosting TNF, IL-6, and IL-1β production, without survival advantage (Cui et al., 2023; Pellon et al., 2025). Conversely, live commensal *Lactiplantibacillus plantarum* induces a tolerogenic training program: gut macrophages produce less TNF, IL-1α/β, IL-6, and CCL20 and fewer antimicrobial peptides (S100A8/9), yet display greater phagocytic activity with reduced reactive-oxygen-species output (Nobs et al., 2020; Pellon et al., 2021).

Impact of enteric helminths in shaping gut-trained immunity has been shown in a number of studies. For example, antigens from *Trichuris suis* promote oxidative phosphorylation without increasing glycolysis, while yielding macrophages that suppress IL-6 and TNF secretion (Zakeri et al., 2022). Lung-migrating parasites such as *Nb* and *Heligmosomoides polygyrus (Hp*) reprogram BM progenitors and regional macrophages, skewing toward M2 functions that accelerate tissue repair and bolster type-2 responses (Netea et al., 2020; Pellon et al., 2025; Negi et al., 2019; Jeyanathan et al., 2022; Silva et al., 2022; Stražar et al., 2021).

Nutritional triggers have also been shown to have critical impacts on trained immunity of gut macrophages, especially those induced by dietary interventions such as starvation as well as selective elimination of food products (Nobs et al., 2020; Christ et al., 2018; David et al., 2014; Napier et al., 2019; Seufert et al., 2022). It has been observed that western diet, as opposed to standard chow diet for mice, causes an increase in circulating proinflammatory cells and cytokines via NLRP3 signaling (Christ et al., 2018). On the other hand, resveratrol has been shown to enhance BCG-induced trained immunity of macrophages causing reduction in the production of pro-inflammatory cytokines such as IL-6 and TNF upon LPS stimulation promoting a tolerogenic phenotype (Pellon et al., 2025; Bulut et al., 2025). In models of colitis, mice pre-treated with β-glucan have shown contrasting results. Treatment with β-glucan 14 days prior to induction of colitis using dextran sulphate sodium (DSS) had worsened symptoms with increased inflammation compared to controls (Del Gaudio et al., 1989; Hajishengallis et al., 2019). However, β-glucan treatment given 26 days prior to induction of colitis showed an improved colonic architecture and lowered myeloperoxidase levels (Hajishengallis et al., 2019; Zhang et al., 2017).

Despite these advances, major questions still persist: (i) how bidirectional interactions between microbiota and macrophage training are regulated; (ii) which specific microbial species most effectively induce protective trained immunity; and (iii) how microbiota-derived metabolites shape durable myeloid memory in the gut and distant organs such as the lung and brain. Addressing these gaps will yield new therapeutic avenues for IBD, food allergies, and other diet-linked inflammatory disorders.

### Brain

Microglia is the brain’s long-lived resident macrophages are well positioned to develop trained-immunity programs. Repeated systemic LPS exposure results in elevated blood cytokine levels and subsequent morphological changes in the microglia and astrocytes (Wendeln et al., 2018). Additionally, primary hippocampal microglia from *S. typhimurium*-infected mice show an increase in CD11c and MHCII expression following LPS challenge one month later (Püntener et al., 2012), indicating that microglial priming rather than tolerance contributed to accelerated neurodegeneration, as shown in Alzheimer’s disease (AD). In studies with neonatal mice treated with LPS, increased microglial IL-1β production resulted in decreased neurogenesis in hippocampus resulting in memory impairments (Bilbo et al., 2005; Bland et al., 2010; Lajqi et al., 2020).

**Figure 4.**
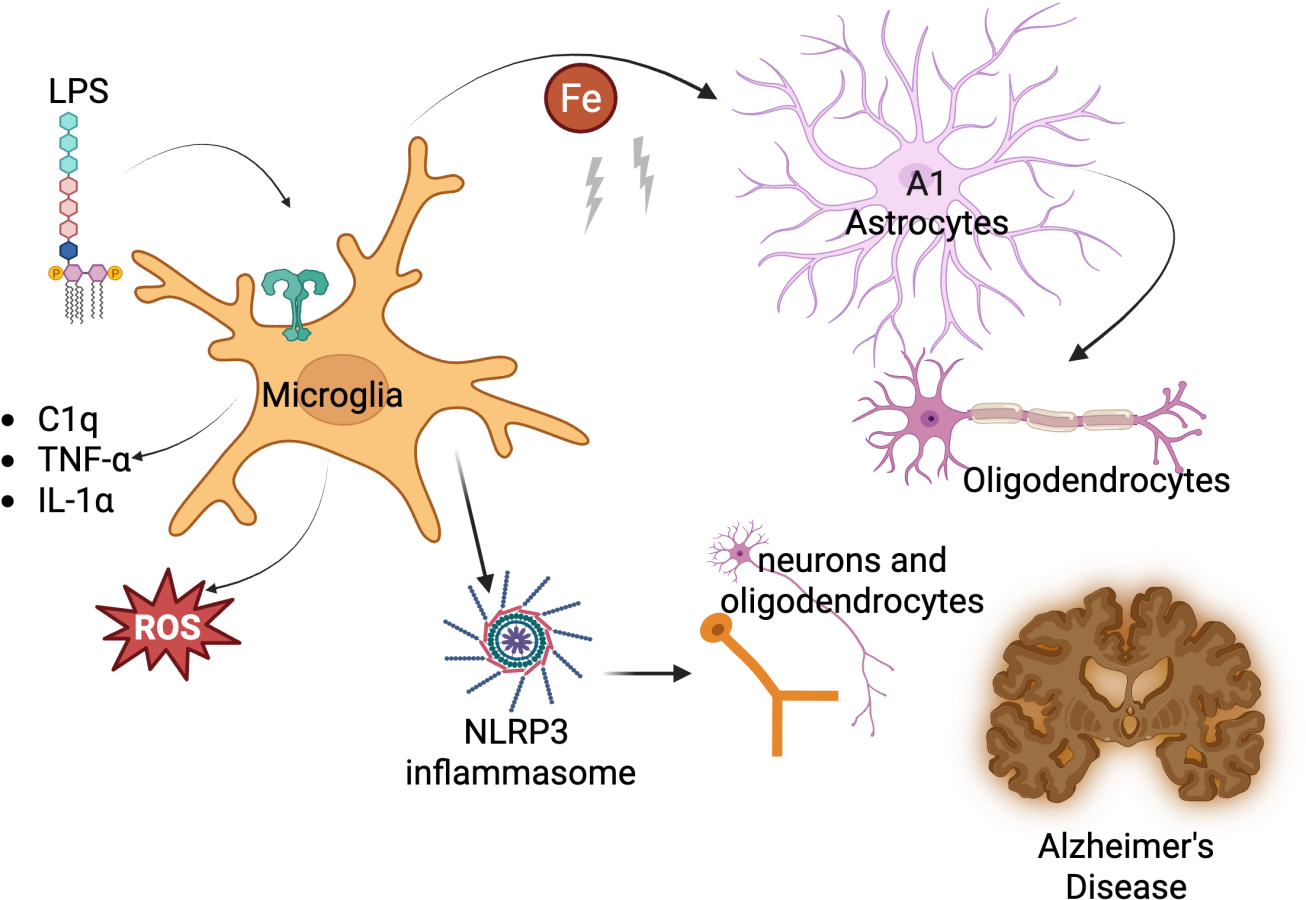
Trained immunity in microglia and its role in Alzheimer’s Disease. Lipopolysaccharide (LPS) activates microglial Toll-like receptors (TLRs), triggering the release of C1q, TNF-a, and IL-1a, which activate A1 astrocytes that damage neurons and oligodendrocytes, contributing to Alzheimer’s pathology. LPS-induced ferritinophagy causes an increase in intracellular iron (Fe), leading to ROS production and NLRP3 inflammasome activation, driving chronic inflammation. This inflammatory response may override microglial tolerization, promoting neurodegenerative disease, while reducing training could mitigate neuronal loss. Image generated using BioRender.com.

Furthermore, activated microglia induce neurotoxic reactive astrocytes via secretion of IL-1α, TNF-α, and C1q (Liddelow et al., 2017). Subsequent TLR engagement drives microglial iron sequestration and triggers AD-like pathology (Zeineh et al., 2015), while anti-C1q antibodies are now being explored clinically to counter this cascade (Lansita et al., 2017). Age-related chronic inflammation exacerbates intracellular iron accumulation, activating the NLRP3 inflammasome and NF-κB pathways that promote neuronal loss (Song and Kim, 2018). The resulting interplay of iron dysregulation, reactive oxygen species, and microglial priming initiates and fuels AD progression, as shown in Figure 4, which is modified from a version adapted from Sfera et al., 2018.

## Therapeutic strategies of targeting trained immunity of macrophages in infections and disease

Recent breakthroughs in medicine and public health have markedly reduced the global toll of infectious and immune-mediated diseases. Nevertheless, stark disparities remain: millions lack reliable access to life-saving therapies, while region-specific threats malaria, tuberculosis, helminth infections, HIV, metabolic disorders, and cancer and the specter of new pandemics continue to exact a heavy price. Because innate immunity governs the earliest phase of host defense, harnessing its memory potential could offer an affordable, broadly protective countermeasure. Long-lived, tissue-resident macrophages exemplify this opportunity; as ‘sentinels, warriors, and healers,’ they patrol virtually every organ and stand ready to mount a rapid, context-appropriate response (Bernier et al., 2025).

**Figure 5.**
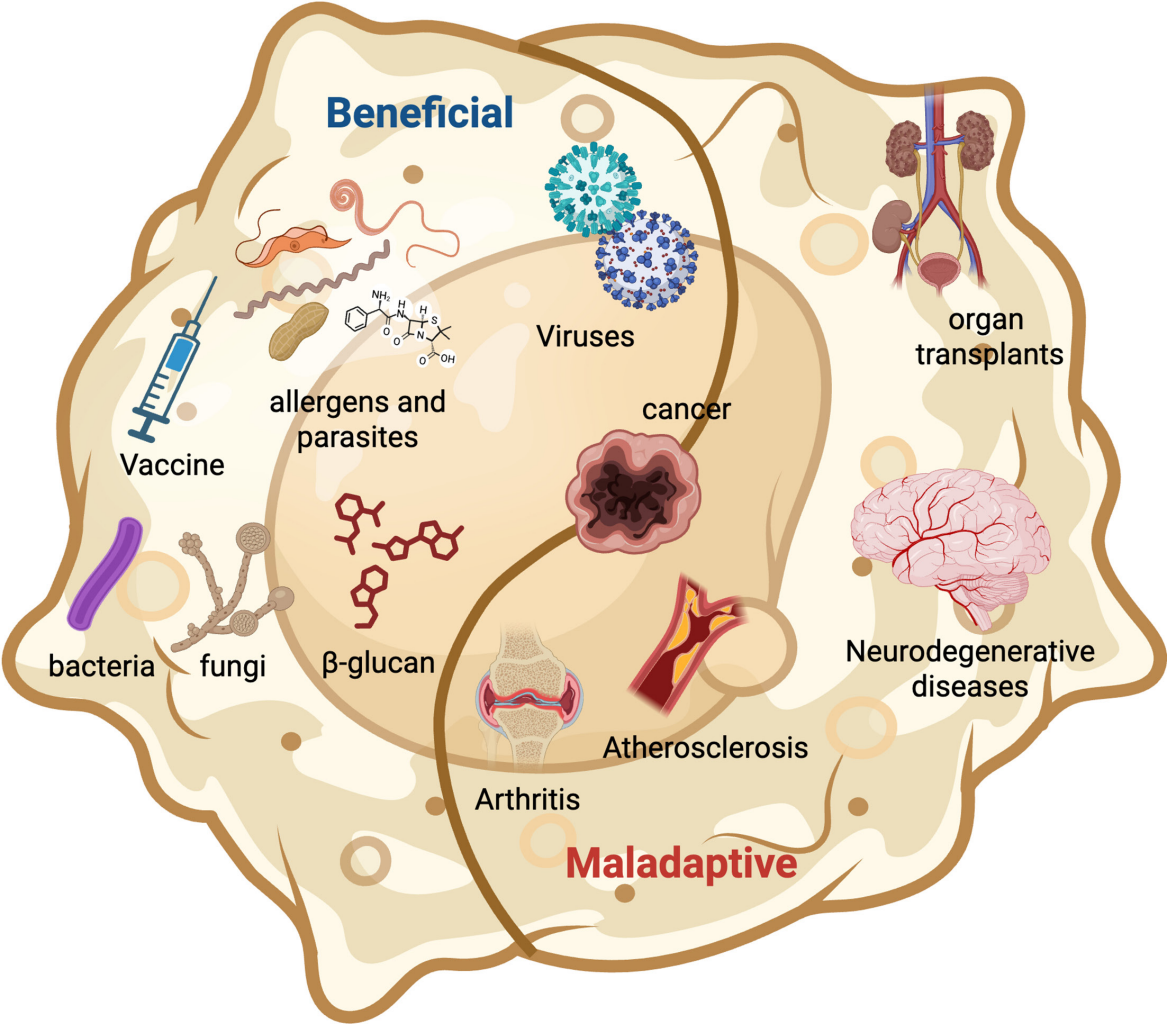
Consequences of trained immunity- host beneficial and maladaptive. Trained immunity can be advantageous when enhancing host defense and disease outcomes after vaccination, β-glucan therapy, and viral, bacterial, or fungal infections, and even after exposure to select allergens or pollutants. Yet the same innate memory programs may turn maladaptive, exacerbating graft rejection, promoting atherosclerosis, fueling neurodegeneration, and driving autoimmune conditions such as rheumatoid arthritis. In case of cancer, consequences of trained immunity are context-dependent with leading to host-beneficial or host-detrimental outcomes. Image generated using BioRender.com

Trained immunity in macrophages is a double-edged sword. In acute infections, the heightened antimicrobial functions induced by a prior stimulus such as BCG vaccination can provide non-specific protection against heterologous pathogens, bolstering host defense against *Mycobacterium tuberculosis* and beyond. Yet in chronic settings like cancer or autoimmune disease, the same amplified responsiveness may become maladaptive, driving persistent inflammation or dampening cytotoxic anti-tumor immunity. Future therapies must, therefore, calibrate this ‘yin and yang,’ amplifying the protective facets of trained immunity while curbing its pathological excesses (Figure 5). In the next sections, we will thoroughly describe how trained immunity can be instrumentalized for host protection in the context of infectious diseases, cancer, neurodegenerative diseases and autoimmunity.

## Targeting infectious diseases

### Vaccines and adjuvants in therapy

Conventional vaccines generate long-lived, antigen-specific memory in B and T cells. Increasingly, however, prophylactic and therapeutic vaccines are also recognized as potent inducers of trained immunity in monocytes and macrophages, broadening protection to heterologous infections and even some malignancies. The most extensively studied examples include BCG, oral poliovirus (OPV), measles–mumps–rubella (MMR), influenza, vaccinia, and more recently, SARS-CoV-2 vaccines (Netea et al., 2016; Cirovic et al., 2020; Netea and Joosten, 2024a; Bulut et al., 2025; Angulo et al., 2025; Debisarun et al., 2021; Ziogas et al., 2023; Joseph, 2022).

Beyond the protection against tuberculosis (TB) infection, BCG vaccination has been reported to confer nonspecific immunity against heterologous infections, suggesting a multifaceted benefit of vaccination via innate immune memory. Other studies have reported that AMs trained by BCG increase their glycolytic metabolism in a T cell–derived IFNγ–dependent manner (Kaufmann et al., 2018; Yao et al., 2018). Influenza and LPS-induced AM training appears to rely on glucose and fatty acid oxidation. Additionally, LPS-induced training enhances efferocytosis in AMs via increased arginine metabolism, improving inflammation resolution (Chen et al., 2022a; Schilperoort et al., 2023; Chakraborty et al., 2023). In vitro studies on sputum-derived AMs from BCG-vaccinated humans revealed decreased CD11b and HLA-DR expression (Koeken et al., 2020), suggesting metabolic reprogramming and altered immune function (Jeyanathan et al., 2022).

In mice, BCG vaccination guards against secondary infections of *Bordetella pertussis*, *Candida albicans*, *Schistosoma mansoni*, influenza, and SARS-CoV-2 (Kaufmann et al., 2018; O’Neill and Netea, 2020; Spencer et al., 1977; Covián et al., 2019; Nascimento et al., 2008; Pittet et al., 2023; Gillard et al., 2022; Tribouley et al., 1978; van ’t Wout et al., 1992), while in humans it lowers the incidence of multiple unrelated infections (Biering-Sørensen et al., 2017; Rieckmann et al., 2017; Jensen et al., 2006; Lund et al., 2015; Benn et al., 2013; Kleinnijenhuis et al., 2014; Cime-Castillo et al., 2018; Walk et al., 2019; Stewart and Levine, 2011; Powles et al., 1977; Villumsen et al., 2009; Buffen et al., 2014). When administered at birth, BCG cuts neonatal deaths from infectious causes by ~43% yet leaves non-infectious mortality unchanged evidence that cross-protection stems from trained immunity rather than disease-specific effects (Biering-Sørensen et al., 2017). Co-administration of BCG and smallpox vaccines further reduces HIV-1 acquisition risk, underlining its capacity to bolster innate defences (Rieckmann et al., 2017; Jensen et al., 2006). The effect, however, is context-dependent; in a controlled malaria challenge BCG worsened pathology, showing that heightened innate reactivity can sometimes be maladaptive (Walk et al., 2019). Live-attenuated oral polio vaccine (OPV) not only prevents poliomyelitis but also lowers all-cause infant mortality, mitigates morbidity from subsequent influenza infection, and reduces pathology in inflammatory-bowel-disease models (Ziogas et al., 2023; Aaby and Benn, 2019; Chumakov et al., 1992; Upfill-Brown et al., 2017). Likewise, MMR vaccination decreases non-measles mortality and has markedly cut childhood hospitalizations across Europe (Sørup et al., 2014), in part through functional and metabolic reprogramming of non-conventional γδ T cells (Röring et al., 2024).

In addition to BCG vaccinations, other respiratory virus vaccines such as seasonal influenza and COVID-19 shots may impart comparable innate imprinting (Gu et al., 2023; Debisarun et al., 2021). Influenza vaccines have been shown to confer non-specific protection via trained immunity in several contexts. A prospective study demonstrated that seasonal influenza vaccination reprograms monocytes toward heightened cytokine production and enhanced pathogen recognition, correlating with reduced all-cause respiratory mortality (Debisarun et al., 2021; Principi and Esposito, 2024). Furthermore, influenza vaccination has been shown to induce long-lasting transcriptional changes in blood monocytes, boosting responses to unrelated respiratory pathogens (Apps et al., 2024; Hjelholt et al., 2023). A quadrivalent inactivated influenza vaccination has been shown to correlate with lower COVID-19 incidences, (Debisarun et al., 2021), while a trivalent influenza vaccine is associated with lower mortality among COVID-19 patients in Brazil (Fink et al., 2020). Influenza vaccination has pleiotropic effects in reducing the risk of cardiovascular diseases in patients with atherosclerosis as well as other cardiovascular diseases (Macintyre et al., 2013; Barnes et al., 2015; Gurfinkel et al., 2004; Gurfinkel et al., 2002) as well as reducing risks and pathology of type-1 diabetes (Bluestone et al., 2021; Watanabe, 2011).

During the COVID-19 pandemic, most of the world received one of four vaccine platforms viral-vector, mRNA, whole-inactivated, or protein-subunit formulations. Among them, mRNA vaccines drive lasting transcriptional and metabolic reprogramming in monocytes and macrophages, boosting glycolytic flux and antiviral gene signatures (Brueggeman et al., 2022; Gu et al., 2023; Li et al., 2022a; Murphy et al., 2023; Mantovani and Netea, 2020). In addition to strong humoral and T-cell immunity against SARS-CoV-2, researchers have examined whether these and other licensed vaccines elicit cross-protective trained immunity (Dangi et al., 2021). Work in this area has assessed both the capacity of heterologous vaccines (e.g. BCG) to mitigate COVID-19 and the potential of COVID-19 vaccination to enhance innate memory in contexts such as cancer therapy (Buffen et al., 2014; Netea et al., 2023; Chen et al., 2023a; Jurado et al., 2025). A recent comprehensive review by Netea et al., 2023 summarized these findings and concluded that, although BCG, MMR, and Shingrix provide broad nonspecific benefits, they do not generate trained immunity sufficient to protect against SARS-CoV-2. In contrast, the novel COVID-19 vaccines do induce durable macrophage training, which likely contributes to their long-term protective efficacy (Netea et al., 2023). Collectively, these studies establish the critical importance of vaccination-induced trained immunity for protection against infectious diseases.

### Cytokines in therapy

Cytokines are pivotal drivers of trained immunity, steering macrophage and monocyte reprogramming through coordinated epigenetic and metabolic changes. Among them, IL-1β stands out as a master regulator: by inducing durable chromatin remodeling in hematopoietic progenitors and mature monocytes, it boosts myelopoiesis and sustains pro-inflammatory responses in both mice and humans (Mitroulis et al., 2018; White et al., 2020) – a process observed in models of *Mycobacterium tuberculosis* (*Mtb*) infection as well as BCG vaccination (Kaufmann et al., 2018). Additionally, IFN-γ enhances monocyte and macrophage training by augmenting antimicrobial responses; in *Listeria monocytogenes* (*Lm*) infection models, prior IFN-γ exposure led to improved pathogen clearance upon reinfection (Netea et al., 2016).

Type 2 cytokines such as IL-4, IL-5, and IL-13 that are essential for activation and expansion of eosinophils and M2 macrophages mediate tissue repair in the lung after *Nb* infection (Hartung and Esser-von Bieren, 2022; Yasuda et al., 2018; Ahrends et al., 2021; Turner et al., 2018). IL-4 in particular drives innate memory in macrophages by promoting an anti-inflammatory, wound-healing phenotype, particularly in helminth infections and allergic inflammation (Czimmerer et al., 2022; Gocheva et al., 2010; Rolot and Dewals, 2018; Netea et al., 2015; Bouchery et al., 2020). Prolonged IL-4 exposure results in sustained STAT6 phosphorylation, a key transcription factor in IL-4 signaling, suggesting that continued stimulation may induce lasting epigenetic changes in macrophages (Ishii et al., 2009). IL-4-polarized macrophages undergo significant epigenomic remodeling and display an amplified inflammatory response upon subsequent LPS (Bukhbinder et al., 2022) challenge. This phenomenon, termed ‘extended synergy,’ is characterized by increased enhancer activity and expansion of the NF-κB-p65 cistrome, indicating that IL-4-induced epigenetic reprogramming primes macrophages for heightened secondary responses (Czimmerer et al., 2022). Moreover, primary infection with *Hp* parasites has been shown to attenuate experimental autoimmune encephalomyelitis (EAE) in an IL-4Ra-dependent manner, suppressing IL-17A secretion. IL-5 also contributes to trained immunity by reprogramming of BM progenitors following a primary allergen exposure altering hematopoiesis and granulopoiesis (Hartung and Esser-von Bieren, 2022). This may drive type 2 inflammation in asthmatic patients through the expansion of CD34^+^ progenitors at inflammatory sites. IL-13 has been implicated in disrupting epithelial barrier function and promote chronic type 2 airway inflammation via reprogramming of epithelial cells (Hartung and Esser-von Bieren, 2022; Hartung and Esser-von Bieren, 2022; Ordovas-Montanes et al., 2018; Ordovas-Montanes et al., 2020; Lundahl et al., 2022). Among other type 2 cytokines, IL-10 is a known anti-inflammatory cytokine and has been shown to suppress trained immunity in monocytes via STAT3 signaling pathway by preventing increased ROS production in BCG-induced training but did not reverse b-glucan-induced metabolic changes and phagocytosis (Röring et al., 2025). Taken together, administration of recombinant cytokines is a viable and effective option to emulate training via type 1 or type 2 pathogens, thereby inducing a robust immune response against secondary challenges.

### Toll-like receptor agonists

A variety of Toll-like receptor (TLR) agonists have been shown to induce trained immunity in both mouse models and human studies. These include ligands for TLR4 (lipopolysaccharide, LPS), TLR2 (Pam3CSK4), TLR3 (Poly I:C), and TLR5 (flagellin), all of which enhance innate immune responses by promoting metabolic and epigenetic reprogramming in monocytes and macrophages (Foster et al., 2007; Owen et al., 2020; Novakovic et al., 2016; Lachmandas et al., 2016; Ifrim et al., 2014; Van der Heijden et al., 2018; Bekkering et al., 2017). Since the initial evidence of TLR4-induced chromatin modifications in LPS model by Foster et al., 2007, recent studies have shown that specific TLR agonists such as MALP-2 (TLR2 agonist) and CpG (TLR9 agonist) induce histone acetylation of poised macrophage enhancers leading to faster and robust responses upon secondary challenge (Ostuni et al., 2013) as extensively reviewed by Alexopoulou and Irla, 2025.

An in vitro infection of murine BMDMs isolated from TLR2-, TLR3- TLR4-, TLR7-, TLR9-, TLR2/TLR4-, and TLR2/TLR4/TLR9- deficient mice and cultured with *Mtb* showed that TLR9 was critical for protection against infection via FLT-3 ligand-generated DCs. Moreover, heat-killed *M. tuberculosis* (HKMtb) induces trained immunity in human monocytes through HIF-1α and Syk-dependent epigenetic reprogramming, enhancing inflammatory responses to TLR4 (Bukhbinder et al., 2022) and TLR7/8 (R848) but not TLR2/TLR1 or TLR9 ligands, and in vivo, systemic or intranasal HKMtb administration augments pro-inflammatory responses to heterologous LPS challenge in mice (Alexopoulou and Irla, 2025; von Meyenn et al., 2006).

Other PRRs, such as nucleotide-binding oligomerization domain (NOD) receptors (NOD1) activation induces glycolytic programming in human monocyte-derived macrophages in both in vitro as well as murine in vivo models, though with minimal secretion of cytokine production (Murugina et al., 2020). Conversely, NOD2, which is activated by BCG administration, not only contributes to trained immunity via peptidoglycan sensing but also negatively regulates macrophage function through a TNFα-dependent mechanism (Ifrim et al., 2014; Chauvin et al., 2023). These studies exemplify how trained immunity in macrophages and monocytes is regulated by both surface and cytosolic PRRs, offering potential avenues for clinical applications aimed at enhancing immune responses.

Heme, an iron-containing molecule essential for oxygen transport, energy production, antioxidant defense, and signal transduction, is an agonist for TLR4 and has been shown to trigger trained immunity (Sawicki et al., 2015). Heme-induced training in macrophages occurs via IL-1β production, mediated by LPS recognition and activation of the NLRP3 inflammasome, leading to enhanced protection against bacterial infections (Dutra et al., 2014; Bozza and Jeney, 2020; Jentho et al., 2021). While heme-induced trained immunity has demonstrated protective effects during early stages of bacterial sepsis, it can also exacerbate inflammation, contributing to endotoxic shock under certain conditions (Jentho et al., 2021). These dual effects underscore the importance of context and regulation in harnessing trained immunity for therapeutic benefit. Collectively, TLR-driven trained immunity emerges from diverse ligands that reprogram monocytes and macrophages through epigenetic and metabolic rewiring creating a poised state that enhances protective responses yet carries context-dependent risks of hyperinflammation.

## Targeting trained immunity in cancer

Trained macrophages can be either allies or adversaries in cancer, depending on their activation state and the tumor microenvironment (Steinbach et al., 2024). As discussed earlier, stimuli such as β-glucan and BCG induce trained immunity that skews macrophages toward a proinflammatory or protective phenotype, thereby boosting tumor surveillance and clearance (Chatzis et al., 2025; Theobald et al., 2024; Buffen et al., 2014; Chen et al., 2023a; van Puffelen et al., 2020). This ‘central’ training begins in BM progenitors and culminates in an inflammatory macrophage program. In an orthotopic model of pancreatic ductal adenocarcinoma, for example, β-glucan traffics to the pancreas and drives a CCR2-dependent influx of trained monocytes and macrophages, enhancing cytotoxic activity and slowing disease progression (Geller et al., 2022). Strategically harnessing such central training to push macrophages whether in the lung, pancreas, or other sites toward a tumoricidal profile could, therefore, improve outcomes in both preclinical studies and cancer patients.

After COVID-19 mRNA vaccination, the ensuing innate activation has been linked both to better responses to immune-checkpoint blockade and, in some settings, to transient systemic inflammation that may accelerate tumor growth (Fendler et al., 2022; Chen et al., 2021). On the other hand, administration of both β-glucan and BCG has recently been shown to induce trained immunity and protect against a subsequent tumor challenge in a mouse model of bladder cancer (Jurado et al., 2025; van Puffelen et al., 2020; Ding et al., 2023; Li et al., 2025) suggesting that β-glucan is a safe and effective adjuvant to enhance BCG immunotherapy in solid tumors. These contrasting outcomes mirror observations with influenza and SARS-CoV-2 vaccines: beyond their antigen-specific protection, each can imprint BM-derived and lung-resident macrophages with BCG-like innate memory, sometimes enhancing host defense, sometimes proving maladaptive in immunocompromised or elderly individuals. Vaccine adjuvants add a further layer, as several have now been shown to epigenetically reprogram macrophages and confer durable resistance to secondary infections (Lee et al., 2022; Wimmers et al., 2021).

Influenza infection in mouse models has been shown to induce trained immunity in AMs to exert long-lasting and tissue-specific antitumor immunity, where trained AMs infiltrate into lung tumor lesions and enhance phagocytic and tumor cell cytotoxic functions via epigenetic and metabolic resistance to the tumor-induced immune suppression. This antitumor ability of AMs is dependent on IFNγ and NK cells (Wang et al., 2023). Conversely, trained tumor-associated macrophages (TAMs) may adopt an immunosuppressive phenotype, supporting tumor growth by promoting angiogenesis, suppressing T-cell responses, and facilitating metastasis through secretion of regulatory cytokines (IL-10, TGF-β) (Kalafati et al., 2020). Epigenetic marks such as increased H3K4me3 in inflammatory gene promoters result in persistent monocyte/macrophage priming, fueling chronic tumor-associated inflammation. Many solid tumors, such as breast, lung, and pancreatic cancers, exploit macrophage training mechanisms to establish an immunosuppressive TME. Repeated exposure to tumor-derived signals, such as metabolic byproducts (lactates from tumor glycolysis), trains macrophages and induce an immunosuppressive phenotype (Biswas and Mantovani, 2010). Subsequently, TAMs suppress cytotoxic T cell responses through the production of Arg1, programmed death-ligand 1 (PD-L1), and IL-10, allowing tumors to evade anti-tumor immunity (Gordon and Martinez, 2010). A recent study described an IL-4 signaling axis in BM-derived from basophils and eosinophils that drive pro-tumorigenic myelopoiesis in non-small cell lung cancer (NSCLC) model causing immunosuppressive myelopoiesis in cancer (LaMarche et al., 2024). Accordingly, pairing immune-checkpoint blockade with therapies that selectively inhibit M2-like, and other regulatory TAM subsets may provide a powerful, synergistic strategy for cancer treatment.

Beyond classical activation, trained programs may rewire macrophage phagocytosis by cargo, a lever with direct relevance in tumors. β-glucan-induced training has been reported to spare ADCP while dampening efferocytosis of apoptotic neutrophils, a combination predicted to (i) preserve antibody-mediated tumor cell clearance yet (ii) limit the accumulation of pro-resolving, immunosuppressive mediators derived from apoptotic-cell uptake within the TME. These cargo-specific effects could be harnessed to tilt TAM function toward antitumor immunity and should be considered when pairing β-glucan/BCG with opsonizing antibodies or checkpoint blockade (Chatzis et al., 2025; Li et al., 2025).

## Targeting trained immunity in neurodegenerative diseases

**Figure 6.**
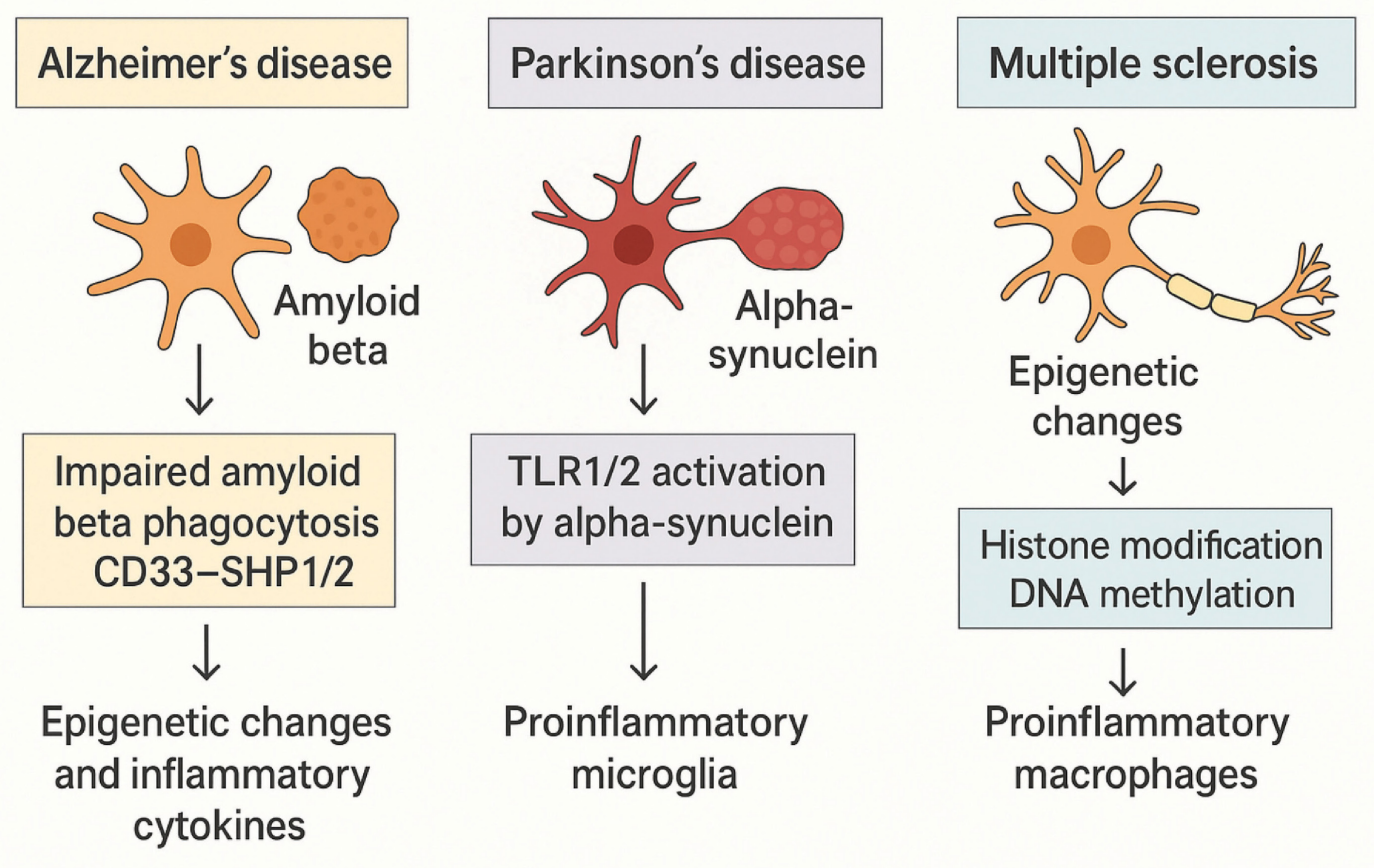
Maladaptive consequences of trained immunity in neurovegetative diseases. In Alzheimer’s disease, trained immunity results in impaired amyloid-β phagocytosis by microglia due to CD33-SHP1/2 signaling, leading to the accumulation of inflammatory cytokines and exacerbation of disease progression. In Parkinson’s disease, the aggregation of α-synuclein activates TLR1/2 receptors on microglia, promoting pro-inflammatory responses that contribute to neurodegeneration. In multiple sclerosis, epigenetic changes, including histone modification and DNA methylation, drive the activation of pro-inflammatory macrophages, further contributing to demyelination and disease progression. The diagram highlights the pathological mechanisms driving these neurodegenerative diseases and underscores the potential role of trained immunity in their progression.

Trained immunity, while advantageous against many infections, can prove harmful in neurodegenerative disorders such as Alzheimer’s disease, Parkinson’s disease, and multiple sclerosis. Unlike systemic macrophage training, which originates in hematopoietic progenitors microglial training unfolds entirely within the brain. The resulting epigenetic imprints persist for years, rendering microglia hyper-responsive and thereby increasing the brain’s vulnerability to chronic inflammation and progressive neurodegeneration (Netea and Joosten, 2024a). Microglial priming is defined as the process of trained immunity in the brain (Haley et al., 2019). In Alzheimer’s disease, trained immunity can worsen pathology. When CD33 on microglia binds sialylated ligands, it recruits the phosphatases SHP-1 and SHP-2, which dampen signaling pathways required for efficient amyloid-β phagocytosis (Griciuc and Tanzi, 2021; McAlpine et al., 2021). Additionally, LPS-stimulated microglia have been shown to cause healthy neuronal death via secretion of TNF, IL-1, and C1q (Sfera et al., 2018). It is now well accepted that epigenetic factors, such as DNA demethylation as well as histone modifications in the promoter region of amyloid precursors cause amyloid beta aggregation in elderly brain causing AD progression (Sanchez-Mut and Gräff, 2015; Liu et al., 2018). In the early stages of AD, increased Ab production and upregulation of inflammatory cytokines have been found to correlate with onset. While in later stages of AD, progression correlates with trained tolerance (Salani et al., 2019; Mishra et al., 2022). Hence, trained immunity of microglia has been correlated with detrimental effects and increased progression of AD. Figure 6 highlights three paradigms in which maladaptive trained immunity fuels neuroinflammation, providing additional evidence that therapeutically targeting macrophage-training pathways could offer neuroprotection in degenerative diseases.

This figure was generated using ChatGPT (GPT-5) on February 10, 2025, and is released under a perpetual, worldwide, royalty-free license permitting unrestricted scientific publication, distribution, and modification.

Additionally, influenza vaccination has also been shown to reduce neuroinflammation in degenerative diseases such as AD. In a large clinical study involving 1,185,611 influenza-vaccinated patients exhibited lower risk of dementia compared to 1,170,868 unvaccinated patients (Bukhbinder et al., 2022). Influenza infection is linked to poorer survival in lung cohorts (Chen et al., 2019; Chen et al., 2022b), highlighting the value of annual influenza vaccination, particularly for patients receiving immune-checkpoint inhibitors to lower risk and improve outcomes (Chong et al., 2020; Failing et al., 2020). These clinical observations echo experimental data showing that a primary influenza infection induces macrophage-mediated trained immunity, which in turn protects against secondary bacterial pneumonia (Aegerter et al., 2020).

Parkinson’s disease (Barriales et al., 2021), an adult-onset neurodegenerative disorder is defined by progressive loss of dopaminergic neurons and the accumulation of α-synuclein–rich Lewy bodies, which drive both its motor and non-motor symptoms (Barriales et al., 2021; Mishra et al., 2022; Dickson, 2025; Schneider et al., 2017). Extracellular α-synuclein is a key driver of Parkinson’s disease that binds TLR1/2 on microglia, activating the TAM-family kinases Mer and (Kim et al., 2013; Fourgeaud et al., 2016; Akalu et al., 2017; Bosurgi et al., 2017). These receptors, which also orchestrate phagocytosis of expendable spinal motor neurons, initiate a pro-phagocytic, inflammatory program that can exacerbate neurodegeneration. Inhibiting Mer/Axl signaling or blocking α-synuclein TLR1/2 engagement may, therefore, attenuate maladaptive microglial training and offer neuroprotective benefit in PD (Hanke and Kielian, 2011).

Multiple Sclerosis (MS) is an inflammatory neurodegenerative disorder that affects the brain and the spinal cord, where the immune system attacks myelin, thus disrupting nerve signals. MS is a lifelong condition with symptoms such as fatigue, numbness, physical imbalance as well as problems with vision, muscles, and cognition (Filippi et al., 2018). Among the triggers for MS, histone modifications such as deacetylation of oligodendrocyte in MS-associated cells (He et al., 2018) as well as histone citrullination in arginine of H3K9me3 preventing secretion of IL-8 and TNF (Fuhrmann and Thompson, 2016) have been hypothesized to lead to chronic MS lesions. However, DNA methylation is critical for brain development and hence, lineagespecific ablation of *Dnmt1* in progenitor cells leads to dramatic hypomyelination manifesting in impaired motor coordination (Moyon et al., 2016). Loss of homeostatic microglia (Zrzavy et al., 2017) as well as phenotypic shift to proinflammatory macrophages and their subsequent production of inflammatory cytokines has been linked to progression of MS in both in vivo and in vitro studies (Miron et al., 2013) and slowly expanding lesions in progressive MS (Jäckle et al., 2020).

Controlling maladaptive trained immunity is vital for limiting microglial overactivation and inflammation that drive neurodegenerative diseases. Therapies that selectively dampen this innate memory, whether through cytokine blockade, immune-checkpoint modulation, or targeted small-molecule inhibitors, hold considerable promise. Histone-deacetylase (HDAC) inhibitors are an especially compelling option: by reshaping the microglial epigenome, they can limit amyloid-β and tau accumulation, restore synaptic plasticity, and improve memory in Alzheimer’s disease. Two agents already approved, vorinostat and tucidinostat, act in part by altering microglial acetylation and thus provide practical entry points for translating trained-immunity modulation into neuroprotective therapy (Ding, 2025; Chuang et al., 2009; Wang et al., 2015). Additionally, blocking Axl and Mer kinases on microglia can also be used to target microglial inflammation in neurodegenerative diseases. Selective immune suppression of JAK/STAT pathway via JAK inhibitors may also offer a strategy to attenuate the maladaptive trained immunity in AD and PD (Sunzini et al., 2020). While Duchenne Muscular Dystrophy (DMD) is not a neurodegenerative disease but rather a neuromuscular pathology disorder, dysregulated inflammation via macrophages and monocytes has been linked to its pathology, implicating trained immunity as a maladaptive contributor to disease progression (Chazaud, 2020). DMD is caused by a genetic mutation in the DMD gene on the X-chromosome that encodes dystrophin, which is a membrane-associated cytoskeletal protein (Duan et al., 2021). Mouse models of DMD (mdx mice) have been instrumental in some of the pivotal work that links a nitric oxide synthase transgene in ameliorating the pathology of muscular dystrophy (MD) (Wehling et al., 2001). Mouse experiments have shown that prior to recruitment of monocytes and macrophages to skeletal muscle, they undergo epigenetic modifications in the BM. In vitro stimulation of BMDMs derived from mdx mice induced with MD with TLR agonists show increased expression of both M1 and M2 macrophage markers causing an inappropriate balance between TNF and TGF-β production within the hybrid monocyte/macrophage lineage suggesting evidence of trained immunity causing rapid degeneration (Bhattarai et al., 2022). In conclusion, targeting CTM in DMD might be a viable target in alleviating progression of degenerative pathobiology.

## Targeting trained immunity in autoimmunity

Development of atherosclerosis can also arise as a complication of unresolved autoimmunity in rheumatoid arthritis (RA) patients (Arts et al., 2018; Heinhuis et al., 2011). In RA, synovial macrophages initially exhibit protective roles by producing inflammation-resolving lipid mediators and supporting repair responses in the synovial fibroblasts in vitro (Alivernini et al., 2020). However, persistent activation of macrophages and monocytes contributes to chronic inflammation and tissue damage. These cells are key drivers of cartilage and bone destruction through several mechanisms, including the production of RANKL, upregulation of glycolysis, increased oxygen consumption, and failure to repress pro-inflammatory gene expression due to defective histone methylation at the TNFα and IL6 promoters. Furthermore, hyperactivation of the PI3K/mTOR signaling pathway leads to elevated CD11b expression on CD14^+^ monocytes, which has been associated with reduced responsiveness to anti-rheumatic therapies. These findings underscore the role of trained immunity and epigenetic dysregulation in sustaining pathological inflammation in RA and its cardiovascular comorbidities (Weyand et al., 2017; Arts et al., 2018; Toh et al., 2014; Mulherin et al., 1996; Shirai et al., 2016; Lioté et al., 2003).

Atherosclerotic plaque formation can also emerge from other metabolic diseases such as obesity and diabetes, where macrophages within adipose tissue undergo maladaptive trained immunity, promoting chronic low-grade inflammation through IL-1β and TNF-α production, which contributes to insulin resistance (Hotamisligil and Erbay, 2008). Hyperglycemia and dyslipidemia can induce trained immunity in BM-derived monocytes that infiltrate adipose tissue, where they contribute to insulin resistance (Rohm et al., 2022). Macrophages in adipose tissue of obese hosts undergo metabolic and epigenetic reprogramming, leading to persistent production of IL-1β and TNFα, which disrupt insulin signaling in adipocytes (Hotamisligil and Erbay, 2008). Hence, inflammatory macrophages and monocytes shaped by prior training in the BM can exert detrimental effects across a range of autoimmune and cardiovascular disorders.

Maladaptive trained immunity has emerged as a key contributor to the persistence of chronic inflammation in many other types of autoimmune and immune-mediated inflammatory diseases, such as RA, systemic lupus erythematosus (SLE), multiple sclerosis (MS), psoriasis, inflammatory bowel disease (IBD). In these conditions, monocytes and macrophages undergo epigenetic and metabolic rewiring—including H3K4me3 and H3K27ac enrichment at inflammatory loci, shifts toward glycolysis and cholesterol/mevalonate metabolism, and mitochondrial remodeling—that sustain exaggerated IL-1β, TNF, and IL-6 responses long after the initiating trigger has resolved (Ziogas et al., 2023; Municio and Criado, 2020; Riksen et al., 2023). Both CTM, imprinted at the level of hematopoietic stem and progenitor cells, and PTM, occurring in tissue-resident macrophages, contribute to autoimmune pathology. Central reprogramming drives pro-inflammatory myelopoiesis for months, while local training in tissue macrophages supports recurrent inflammation at barrier sites such as the gut, skin, and synovium. Recent work shows that oral barrier inflammation can export pathology to distal sites by training HSPCs through IL-1R–dependent pathways, thereby exacerbating arthritis susceptibility.

Several mechanistic nodes have been identified as actionable targets. The IL-1/NLRP3 axis is a recurrent driver of maladaptive trained immunity, with preclinical and clinical evidence supporting therapeutic blockade using IL-1 antagonists (anakinra, canakinumab) or small-molecule NLRP3 inhibitors (Municio and Criado, 2020; Riksen et al., 2023). Lipid and mevalonate pathway flux also reinforce TI programs in monocytes, suggesting that statins or other metabolic interventions could mitigate disease activity (Riksen et al., 2023). Similarly, glycolytic and mitochondrial pathways (via AKT–mTOR–HIF-1α) sustain hyperinflammatory phenotypes in RA, SLE, and IBD and can be countered by adenosine monophosphate-activated protein kinase (AMPK) activators such as metformin or by mTOR inhibitors such as rapamycin (Municio and Criado, 2020). Epigenetic interventions—including histone deacetylase (HDAC) or DNA methyltransferase (DNMT) inhibitors—are being explored to reset chromatin accessibility in chronically trained macrophages, although their clinical utility will depend on achieving specificity while minimizing infection risk (Ziogas et al., 2023).

Disease-focused studies underscore the translational potential of these strategies. In RA and spondyloarthritis, combining cytokine blockade (anti-TNF, anti-IL-1) with metabolic modulators such as metformin has been proposed to dampen flare-associated trained responses (Municio and Criado, 2020). In SLE, interferon-driven myelopoiesis and neutrophil extracellular traps reinforce maladaptive TI, pointing again to the IL-1/NLRP3 axis and metabolic pathways as promising intervention points (Ziogas et al., 2023). In MS, trained microglia and monocytes perpetuate demyelinating inflammation, highlighting opportunities for epigenetic and metabolic reprogramming adjuncts to existing lymphocyte-targeted biologics (Ziogas et al., 2023). Likewise, in psoriasis and IBD, repetitive barrier challenges reprogram tissue macrophages toward persistent IL-1β and TNF-α secretion, suggesting that compartment-specific interventions aimed at local macrophage training may complement systemic therapies (Municio and Criado, 2020). Finally, at the cardiometabolic interface, Western diet, hyperlipidemia, and diabetes drive TI in myeloid cells and exacerbate vascular inflammation; interventions such as statins, IL-1 blockade, and metabolic ‘de-training’ approaches could reduce autoimmune flares in patients with comorbid cardiovascular disease (Riksen et al., 2023; Tercan et al., 2021).

Future therapeutic strategies will need to distinguish central from peripheral training and develop compartment-specific approaches. Nanoparticle delivery systems, bioengineering methods, and tolerogenic cell therapies are being investigated to selectively target HSPCs or tissue macrophages, potentially minimizing systemic immunosuppression while resetting maladaptive innate memory (Municio and Criado, 2020). Equally important will be aligning intervention timing with the biology of TI, as peripheral training is often detectable for days to weeks while central training can persist for months, and implementing biomarkers such as ATAC-seq, metabolomics, and cytokine recall assays to track therapeutic impact (Ziogas et al., 2023; Municio and Criado, 2020; Tercan et al., 2021). Together, these advances clarify that targeting TI in autoimmunity is not vague but rests on well-defined molecular pathways and translational strategies, offering new opportunities to recalibrate innate immune memory for durable disease control. Reversing maladaptive trained immunity in chronic inflammatory and autoimmune diseases will likely demand a multifaceted approach. Promising strategies include metabolic reprogramming, epigenetic modulation with HDAC inhibitors, and biologic therapies that neutralize pivotal pro-inflammatory cytokines such as TNF, IL-17, and IL-1β released by activated macrophages and monocytes.

## Concluding remarks

Advances in modern medicine have sharply reduced deaths and disability from infectious and immune-mediated diseases. Yet deep global inequities in care and new challenges such as emerging pandemics, malaria, tuberculosis, and metabolic disorders continue to strain health-care systems. At the same time, our expanding knowledge of trained immunity reveals a double-edged sword: when properly tuned, it offers broad protection, but when dysregulated, it can drive chronic inflammation. Evidence from animal models and early human studies highlights both the promise and peril of trained macrophages and monocytes, particularly in the respiratory tract. Progress, however, is limited by gaps in experimental tools: we lack models that can selectively ablate specific tissue-resident macrophage subsets, and the cells’ plasticity makes it hard to distinguish ‘central’ (BM-derived) from ‘peripheral’ (local) training. While key epigenetic modifications, including H3K4me3, H3K27ac, H3K9me3, and DNA methylation have been characterized, the exact sequence of epigenomic events driving macrophage reprogramming remains unresolved. Similarly, the role of non-coding RNAs (lncRNAs and miRNAs) in shaping trained immunity is largely unknown. Resolving these issues will be critical for manipulating trained immunity for improved immunotherapy and vaccine design.

The duration of trained immunity also raises important questions. Human studies suggest that innate memory can persist for up to a year, indicating that long-lived hematopoietic stem cells (HSCs) may serve as reservoirs for epigenetically modified myeloid cells. Yet, the interplay between central and peripheral training and the impact of overlapping macrophage markers in tissues such as the lung, skin, and gut remains poorly defined.

Additional uncertainties include whether trained macrophages can revert to a naïve state, and how extrinsic factors like diet, the microbiome, and chronic infections modulate the stability of trained immunity. The heterogeneity among tissue-resident macrophage subsets (e.g. alveolar, interstitial, Kupffer cells, microglia, and splenic macrophages) further complicates our understanding of their distinct capacities for training and systemic influence.

Moreover, the mechanisms governing macrophage metabolic shifts, especially the switch between glycolysis and oxidative phosphorylation, as well as the roles of fatty acid oxidation and glutaminolysis are not fully defined. Given that metabolic modifiers such as mTOR inhibitors and AMPK activators are already used therapeutically, elucidating these pathways could offer new strategies for cancer, infectious, and metabolic diseases.

Lastly, the impact of aging on macrophage-trained immunity is unclear. Age-related changes in metabolism and epigenetics might predispose macrophages to a pro-inflammatory state, reducing their protective capacity. Addressing these gaps is crucial for developing rejuvenation strategies that enhance immune defenses and improve vaccine efficacy and cancer therapies in older populations.

Targeting trained immunity through vaccines and adjuvants is emerging as a powerful strategy to provide broad, non-specific protection against diverse pathogens. Classic examples include BCG and seasonal influenza vaccines, which bolster host defense not only against their intended microbes but also against unrelated infections, underscoring the growing importance of trained immunity in vaccine efficacy. Trained immunity causes outcomes that are context-dependent: while BCG augments resistance to tuberculosis and some cancers, it can worsen malaria pathology. Likewise, COVID-19 vaccines induce durable metabolic and transcriptional reprogramming in monocytes and macrophages, illustrating the wider potential of innate memory but also revealing complexities that certain vaccine-induced trained responses may amplify inflammation or facilitate tumor progression. Fully harnessing the benefits of trained immunity, particularly for immunocompromised or chronically ill individuals, will depend on dissecting the precise molecular and cellular mechanisms that tip the balance between protection and pathology.
